# Non-Invasive Brain Stimulation for Central Neuropathic Pain: An Exploratory Randomized Sham-Controlled Crossover Trial

**DOI:** 10.3390/brainsci16101078

**Published:** 2026-10-08

**Authors:** Sooyoung Kim, Eun Kyoung Lee, Youngkwon Ko, Hyun-Wook Nah, Hye Seon Jeong, Seong-Hae Jeong, Ae Young Lee, Eunhee Sohn

**Affiliations:** 1 Department of Neurology, Chungnam National University Hospital, Chungnam National University College of Medicine, 282 Munhwa-ro, Jung-gu, Daejeon 35015, Republic of Koreahwnah@cnuh.co.kr (H.-W.N.); mseaj@hanmail.net (S.-H.J.);; 2 Department of Neurology, Chungnam National University Sejong Hospital, Chungnam National University College of Medicine, Sejong 30099, Republic of Korea; 2021eunklee@cnuh.co.kr; 3 Department of Anesthesiology and Pain Medicine, Chungnam National University Hospital, Chungnam National University College of Medicine, Daejeon 35015, Republic of Korea

**Keywords:** central neuropathic pain, repetitive transcranial magnetic stimulation, transcranial direct current stimulation, neuromodulation, non-invasive brain stimulation

## Abstract

**Highlights:**

**What are the main findings?**

- In this exploratory sham-controlled crossover trial in central neuropathic pain, neither high-frequency M1-rTMS nor anodal M1-tDCS produced a statistically significant reduction in pain intensity compared with modality-matched sham stimulation.
- Point estimates for rTMS at 1 week were directionally favourable but imprecise and were attenuated after adjustment for a baseline age imbalance; no difference between rTMS and tDCS was demonstrated.

**What are the implications of the main findings?**

- These data do not establish the efficacy of either modality or the superiority of one modality over the other.
- Adequately powered trials with balanced baseline characteristics, formal assessment of blinding, and longer follow-up are needed to determine whether short-course M1-rTMS provides clinically meaningful benefit.

**Abstract:**

**Background/Objectives:** Central neuropathic pain (CNP) is difficult to manage, and comparative evidence for non-invasive brain stimulation is limited. We explored the short-term efficacy and safety of repetitive transcranial magnetic stimulation (rTMS) and transcranial direct current stimulation (tDCS) in CNP. **Methods:** In this randomized, sham-controlled crossover trial, 26 patients were allocated to active or sham stimulation and received high-frequency rTMS (10 Hz, 2000 pulses/session) and anodal tDCS (2 mA, 30 min) over the primary motor cortex for five days each, separated by a 4-week washout. The primary outcome was change in Numerical Rating Scale (NRS) pain intensity at 1 and 4 weeks, analysed using factorial linear mixed-effects models with and without age adjustment. **Results:** At 1 week, the difference between active and sham rTMS was 0.76 points (95% CI −0.005 to 1.53; *p* = 0.051) and 0.42 points after age adjustment (95% CI −0.37 to 1.21; *p* = 0.30). Active tDCS did not differ from sham tDCS, and active rTMS did not differ from active tDCS. No significant differences were found at 4 weeks or in responder analyses. No serious adverse events occurred. **Conclusions:** In this small exploratory trial, neither rTMS nor tDCS showed statistically significant superiority over modality-matched sham, and no difference between modalities was demonstrated. Adequately powered trials are needed.

## 1. Introduction

Neuropathic pain, defined as pain caused by a lesion or disease of the somatosensory nervous system [1], represents a significant clinical challenge due to its chronicity and limited response to conventional treatments [2]. Among its subtypes, central neuropathic pain (CNP), arising from lesions in the brain or spinal cord, is particularly difficult to manage and is often associated with substantial impairment in quality of life.

CNP is often difficult to manage and is characterized by maladaptive changes within central nociceptive and antinociceptive networks. This may reflect the fact that, in CNP, the lesion is located within the central nervous system itself, where pain persistence is associated with maladaptive neuroplasticity, including central sensitization, cortical reorganization, and altered network-level pain processing. In contrast, peripheral neuropathic pain arises from injury to the peripheral nervous system; although central sensitization can develop secondarily, ongoing peripheral input is thought to contribute to its maintenance, suggesting that earlier control of peripheral nociceptive drive may mitigate subsequent central amplification [3]. Consequently, CNP may be less responsive to conventional treatments and may require therapeutic approaches that more directly target central pain modulatory networks [4,5].

Non-invasive brain stimulation (NIBS) has emerged as a promising neuromodulatory approach for neuropathic pain. Repetitive transcranial magnetic stimulation (rTMS) and transcranial direct current stimulation (tDCS) are the most widely used techniques, both of which modulate cortical excitability and influence pain-related neural networks. High-frequency rTMS applied to the primary motor cortex (M1) has demonstrated short-term analgesic effects in several neuropathic pain conditions [6,7], whereas tDCS offers advantages in terms of simplicity, safety, and cost-effectiveness, although its efficacy appears more variable [8,9]. Although both rTMS and tDCS have been individually investigated in patients with CNP, direct head-to-head comparisons between the two modalities remain scarce. A previous randomized crossover study directly compared high-frequency rTMS and anodal tDCS in patients with drug-resistant neuropathic pain [10]; however, the study included a heterogeneous population comprising both central and peripheral neuropathic pain etiologies and did not incorporate a sham-control condition. Thus, comparative evidence from sham-controlled studies specifically focused on CNP remains limited.

Therefore, the present study was designed to directly compare the short-term analgesic efficacy and safety of rTMS and tDCS in patients with CNP using a randomized, sham-controlled crossover design.

## 2. Materials and Methods

### 2.1. Study Design

This study was a prospective, randomized, sham-controlled crossover trial conducted at Chungnam National University Hospital. The study protocol was approved by the Institutional Review Board of Chungnam National University Hospital (IRB No. 2023-07-061). All participants provided written informed consent prior to enrollment, and the statistical analysis plan is available from the corresponding author upon reasonable request. The study was designed and reported in accordance with the CONSORT guidelines for randomized trials and the CONSORT extension for randomized crossover trials [11,12]. Our clinical study has been registered in the Clinical Research Information Service (CRIS), and the registration number is KCT0012656.

### 2.2. Participants

Patients aged 18 years or older with CNP persisting for at least 3 months were eligible. CNP was diagnosed by a neurologist according to the revised grading system for neuropathic pain (2016), and only patients classified as probable or definite neuropathic pain according to this grading system were included [13]. To ensure diagnostic accuracy, the distribution of pain and sensory abnormalities was assessed for neuroanatomical consistency with lesions in the central nervous system. Detailed clinical history and neurological examination were performed to evaluate the location, extent, and characteristics of pain and sensory disturbances. Only patients whose symptoms were anatomically and clinically consistent with confirmed central lesions were included. Patients with a pain intensity of 4 or higher on the Numerical Rating Scale (NRS), despite stable pharmacological treatment for at least 4 weeks, were included. Medication regimens and dosages were maintained without change throughout the study period. Major etiologies included post-stroke pain and spinal cord lesions. Patients were excluded if they had intracranial metallic implants, implanted electronic devices, a history of epilepsy or syncope, structural brain lesions unrelated to CNP, or were pregnant. Patients receiving medications that could significantly alter cortical excitability were also excluded.

A total of 26 patients who met the inclusion criteria were enrolled between 1 September 2023 and 31 August 2025.

### 2.3. Interventions

All procedure sessions were delivered by a single neurologist with 3 years of experience in administering NIBS. As this was a single-centre trial, no site-level eligibility criteria were applied.

#### 2.3.1. rTMS Protocol

High-frequency rTMS was delivered using a Magstim Rapid2 stimulator with a figure-of-eight coil (The Magstim Company Ltd., Whitland, UK). The motor hotspot was identified without neuronavigation by systematically positioning the coil over the M1, with the coil held at approximately 45° to the midsagittal plane and the handle directed posterolaterally. The motor hotspot was defined as the scalp location at which visible contralateral hand movement could be elicited most consistently at the lowest stimulation intensity. The visual resting motor threshold (vRMT) was then determined at this location and defined as the lowest stimulator intensity that elicited an observable hand movement in at least 5 of 10 consecutive stimuli while the participant was at rest [14].

Therapeutic stimulation was delivered over the identified motor hotspot at 10 Hz and at an intensity of 80% of the vRMT, in accordance with established safety guidelines [15]. Each session consisted of 2000 pulses (20 trains of 100 pulses with an inter-train interval of 80 s), delivered over approximately 30 min. Stimulation was administered once daily for five consecutive days.

#### 2.3.2. tDCS Protocol

Anodal tDCS was delivered using a DC-stimulator Plus device (neuroConn GmbH, Ilmenau, Germany). A constant current of 2 mA was applied for 30 min using saline-soaked sponge electrodes (5 × 7 cm). The anode was positioned over the M1 corresponding to the pain side (C3 or C4 according to the international 10–20 EEG system [16]), and the cathode was placed over the contralateral supraorbital region [17]. Stimulation was administered once daily for five consecutive days.

#### 2.3.3. Targeting of Stimulation

For both interventions, stimulation was applied to the M1 contralateral to the pain side, consistent with established rTMS and tDCS protocols [6,17]. In patients with bilateral pain, the left M1 was selected as the predefined stimulation target.

#### 2.3.4. Sham Stimulation

Sham rTMS was delivered using a sham coil producing a similar acoustic click sound without effective magnetic stimulation. For tDCS, current was applied for 10 s and then ramped down to zero to mimic the initial sensation of active stimulation [18].

### 2.4. Study Procedure

Participants were blinded to the stimulation condition (active vs. sham), whereas the stimulation modality (rTMS or tDCS) could not be blinded because of the inherent differences between the two procedures. Outcome assessments were performed using standardized self-reported measures.

Participants were first randomly assigned in a 1:1 ratio to either the active-stimulation or sham-stimulation condition. Within each condition, participants were further randomized in a 1:1 ratio to one of two intervention sequences: rTMS followed by tDCS or tDCS followed by rTMS. Thus, participants were allocated to one of four treatment sequences: active rTMS followed by active tDCS, active tDCS followed by active rTMS, sham rTMS followed by sham tDCS, or sham tDCS followed by sham rTMS. Randomization was performed by an independent statistician at ACROSS, Chungnam National University Hospital, using computer-generated random sequences. Allocation was concealed using sequentially numbered, sealed, opaque envelopes prepared by a research coordinator who was not involved in participant enrollment or outcome assessment. Participants were enrolled by clinicians, who had no access to the random allocation sequence. After enrolment, participants were assigned to groups by a research coordinator who held the allocation table and was not involved in participant enrolment or delivery of the stimulation. Each intervention period consisted of five consecutive daily stimulation sessions. Outcome assessments were performed at baseline, immediately after completion of the five-session treatment course (approximately 1 week after baseline), and 4 weeks after treatment. Importantly, for the first treatment period, the 4-week assessment was performed immediately before initiation of the second treatment period and therefore also served as the baseline assessment for the second intervention. Thus, pain intensity was reassessed after the 4-week washout period before crossover to the subsequent stimulation modality. A schematic overview of the study design is presented in Figure 1.

Participants were first randomly assigned in a 1:1 ratio to either the active-stimulation or sham-stimulation condition. Within each condition, participants were further randomized in a 1:1 ratio to one of two intervention sequences: rTMS followed by tDCS, or tDCS followed by rTMS. Each intervention consisted of five consecutive daily sessions, followed by a 4-week washout period. Outcome assessments were performed at baseline (T0), immediately after stimulation at 1 week (T1), and 4 weeks after stimulation (T2). The primary outcome was pain intensity (NRS), and secondary outcomes included NPSI, painDETECT, BDI, PSQI, and WHOQOL-BREF. For treatment-aligned analyses, the 4-week assessment after Period 1 was treated as T2 for Period 1 and simultaneously as T0 for Period 2. Treatment satisfaction (patient-reported treatment preference) was assessed at the end of the study.

A total of 26 participants were randomized to either the active stimulation group (*n* = 13) or the sham group (*n* = 13). Participants in the active group received both rTMS and tDCS in a randomized crossover sequence, separated by a 4-week washout period. Participants in the sham group received sham rTMS and sham tDCS in a similar crossover design. One participant in the active group did not complete the tDCS condition because of personal scheduling conflicts that prevented continued participation in the study, resulting in 12 participants included in the tDCS analysis. All participants in the sham group completed both conditions. Final analyses included active rTMS (13 observations), active tDCS (12 observations), sham rTMS (13 observations), and sham tDCS (13 observations), contributed by 26 participants overall. Participant allocation and disposition are summarized in Figure 2.

### 2.5. Outcome Measures

The primary outcome was pain intensity measured using the NRS. Secondary outcomes included validated measures of neuropathic pain characteristics assessed by the Neuropathic Pain Symptom Inventory (NPSI) [19,20] and painDETECT questionnaire [21], mood assessed by the Beck Depression Inventory (BDI) [22], sleep quality assessed by the Pittsburgh Sleep Quality Index (PSQI) [23], and quality of life assessed by the WHO Quality of Life-BREF (WHOQOL-BREF) [24].

Assessments were performed at baseline, immediately after each intervention at 1 week, and 4 weeks after each intervention. At the end of the study [at the 4-week assessment after the second treatment], treatment preference was assessed. There were three response options: rTMS was more helpful, tDCS was more helpful, or no difference. This question was adapted from the Patient Global Impression of Change (PGIC) [25]. However, unlike the standard PGIC, it asks participants to compare the two treatments. We therefore refer to this outcome as patient-reported treatment preference.

#### 2.5.1. Safety Assessment

Adverse events were assessed at each session based on patient-reported symptoms, including headache, dizziness, fatigue, nausea, and worsening of pain. Serious adverse events were monitored throughout the study.

#### 2.5.2. Sample Size Calculation

A prior sample-size calculation was performed based on the primary outcome of change in NRS pain intensity. Assuming a between-group difference of 1.5 points in NRS change, a standard deviation of 2.5, a two-sided α of 0.05, and a statistical power of 80%, 44 participants per group were required. Allowing for an anticipated dropout rate of approximately 10%, the target sample size was set at 50 participants per group, for a total enrollment of 100 participants. However, recruitment ended before the prespecified target sample size was reached, and the final sample size was therefore smaller than originally planned.

#### 2.5.3. Statistical Analysis

Continuous variables are presented as mean ± standard deviation or median with interquartile range, as appropriate, and categorical variables as frequencies and percentages. The primary outcome was the change in NRS pain intensity from baseline to 1 and 4 weeks after stimulation. For each treatment period, ΔNRS was calculated as the NRS score at the start of that period (T0) minus the corresponding follow-up score at T1 or T2; therefore, positive values indicate pain reduction. For Period 2, T0 corresponded to the 4-week assessment of Period 1.

Because each participant contributed repeated observations, treatment effects were analysed using linear mixed-effects models with a random intercept for participant. Consistent with the 2 × 2 factorial crossover design, the models included stimulation condition (active vs. sham), stimulation modality (rTMS vs. tDCS) and the condition × modality interaction, together with treatment period and treatment sequence, as fixed effects. Because the active and sham groups differed in age at baseline, each model was fitted both without and with age (centred at the sample mean) as an additional covariate, and both results are reported. Given the baseline age imbalance and its material influence on the treatment-effect estimates, particular emphasis was placed on the age-adjusted results. Models were fitted separately for ΔNRS at 1 week and at 4 weeks using restricted maximum likelihood. Four model-derived contrasts were estimated: active rTMS versus sham rTMS, active tDCS versus sham tDCS, active rTMS versus active tDCS, and sham rTMS versus sham tDCS. Estimates, standard errors, 95% confidence intervals, and *p*-values are reported. No adjustment for multiple comparisons was applied to the model-derived contrasts. Because none of these comparisons were statistically significant at the unadjusted level, adjustment would not have altered the conclusions; results are therefore presented as estimates with 95% confidence intervals. To aid clinical interpretation, responder analyses were performed for each active condition and its modality-matched sham condition. Percentage pain reduction was calculated as (NRS at T0 − NRS at follow-up)/NRS at T0 × 100, and responders were defined by ≥30% and ≥50% reductions and by a ≥2-point reduction in NRS [25,26]. Proportions were compared using Fisher’s exact test, with 95% confidence intervals for differences calculated using the Newcombe hybrid score method, and mean percentage reductions were compared using Welch’s *t*-test.

Secondary continuous outcomes, including NPSI total and subdomain scores, painDETECT, BDI, PSQI, and WHOQOL-BREF, were analyzed using change scores from the period-specific baseline to the corresponding 1-week and 4-week assessments. Because the two modalities within each randomized group were received by the same participants, comparisons of secondary outcomes were chosen to respect this dependence. Modality-matched active-versus-sham comparisons (active rTMS vs. sham rTMS and active tDCS vs. sham tDCS), which involve independent participants, were performed using the Mann–Whitney U test. Within-participant comparisons between modalities (active rTMS vs. active tDCS and sham rTMS vs. sham tDCS) were performed using the Wilcoxon signed-rank test. All analyses of secondary outcomes, including NPSI and its subscales, painDETECT, BDI, PSQI, WHOQOL-BREF, and patient-reported treatment preference, were considered exploratory. No adjustment for multiple comparisons was applied to these exploratory analyses, and their results should therefore be interpreted with caution. Treatment preference was compared between the active and sham groups using Fisher’s exact test (Fisher–Freeman–Halton test). We also examined whether preference differed by treatment sequence. Safety and tolerability outcomes were summarized descriptively as counts and proportions by stimulation condition; no formal hypothesis testing was performed because of the small number of adverse events.

A two-sided *p*-value < 0.05 was considered statistically significant. Linear mixed-effects models were fitted using the statsmodels package (version 0.15.0) in Python (version 3.12.3), with Wald-type 95% confidence intervals and *p* values. Nonparametric tests and Welch’s *t*-tests for the revised analyses were performed using SciPy (version 1.17.1). Other analyses were performed using SPSS version 27.0 (IBM Corp., Armonk, NY, USA). No interim analyses were planned or performed, and no formal stopping guidelines were prespecified. Recruitment was terminated early because of slower-than-expected enrolment; this decision was based solely on feasibility and was made without examination of outcome data.

## 3. Results

A total of 26 patients were enrolled, with 13 patients randomly assigned to the active stimulation group and 13 to the sham group.

Baseline characteristics are summarised in Supplementary Table S1. Participants in the sham group were younger than those in the active group (56.2 ± 10.4 vs. 64.8 ± 6.9 years, *p* = 0.04). Baseline NRS was similar between groups (6.6 ± 2.6 vs. 6.5 ± 1.4, *p* = 0.6), and there were no significant differences in sex, diagnosis, disease duration, stimulation side, medication, or baseline patient-reported outcomes. CNS demyelinating disease was more frequent in the active group than in the sham group (53.8% vs. 23.1%).

### 3.1. Primary Outcome: Pain Intensity

Descriptive NRS scores for the four stimulation conditions are presented in Table 1. Baseline NRS scores were comparable across conditions (active rTMS: 6.4 ± 1.8; sham rTMS: 6.2 ± 2.8; active tDCS: 6.3 ± 1.9; sham tDCS: 6.5 ± 2.4). At 1 week, the mean NRS reduction was 1.1 ± 1.3 after active rTMS, 0.3 ± 0.8 after sham rTMS, 0.4 ± 1.2 after active tDCS, and 0.0 ± 0.4 after sham tDCS; at 4 weeks, the corresponding values were 1.1 ± 2.3, 0.3 ± 1.3, 0.2 ± 1.6, and 0.6 ± 1.2. All 13 participants completed the rTMS period in each group; one participant in the active group did not complete the tDCS period, leaving 12 active tDCS observations.

The results of the factorial linear mixed-effects models are shown in Table 2. In the unadjusted model (Table 2A), the difference between active and sham rTMS at 1 week was 0.76 points (95% CI −0.005 to 1.53, *p* = 0.051), and that between active and sham tDCS was 0.41 points (95% CI −0.37 to 1.20, *p* = 0.30). After adjustment for age (Table 2B), these estimates were attenuated to 0.42 points (95% CI −0.37 to 1.21, *p* = 0.30) and 0.08 points (95% CI −0.72 to 0.88, *p* = 0.84), respectively. Age was associated with greater pain reduction (0.04 points per year, 95% CI 0.01 to 0.07, *p* = 0.023). Active rTMS did not differ significantly from active tDCS (age-adjusted difference 0.65 points, 95% CI −0.09 to 1.40, *p* = 0.087), the condition × modality interaction was not significant (age-adjusted estimate 0.34, 95% CI −0.71 to 1.39, *p* = 0.53), and the two sham procedures did not differ (0.31 points, 95% CI −0.42 to 1.05, *p* = 0.40).

At 4 weeks, no contrast was statistically significant in either model; in the age-adjusted model, the difference between active and sham rTMS was 0.13 points (95% CI −1.14 to 1.39, *p* = 0.84), and that between active and sham tDCS was −1.14 points (95% CI −2.43 to 0.14, *p* = 0.081). Period and sequence effects were small and nonsignificant in all models.

Additional analyses were performed to assess potential carry-over effects. Among participants who received active rTMS in the first period, the mean NRS score before the second treatment was close to the initial baseline value (6.57 ± 1.90 vs. 6.86 ± 0.90), although five of seven participants remained at least 1 point below their initial baseline score (Supplementary Table S2). The change in pain following tDCS was similar whether tDCS was administered in the first or second period (0.50 ± 1.52 vs. 0.33 ± 1.03). In a sensitivity analysis restricted to the first treatment period, where carry-over could not occur, the estimated 1-week difference between active rTMS and sham rTMS was 0.81 points (95% CI −0.34 to 1.96, *p* = 0.15; Supplementary Table S3). In addition, carry-over was formally tested in the active group by comparing the sum of 1-week ΔNRS across both periods between the two treatment sequences (two-sample *t*-test); the estimated carry-over effect was 0.17 points (95% CI −2.30 to 2.64, *p* = 0.88).

Responder analyses are presented in Table 3. At 1 week, 3 of 13 participants (23.1%) had a pain reduction of at least 30% after active rTMS, compared with none after sham rTMS (*p* = 0.22); the corresponding proportions for a reduction of at least 50% were 15.4% and 0% (*p* = 0.48), and for a reduction of at least 2 points, 38.5% and 15.4% (*p* = 0.38). No responder comparison between active and modality-matched sham stimulation was statistically significant for either modality at 1 or 4 weeks.

### 3.2. Secondary Outcomes

Change scores for secondary outcomes are presented in Supplementary Table S4. No significant differences in NPSI total score were found between active and sham rTMS (*p* = 0.91 at 1 week; *p* = 0.80 at 4 weeks), between active and sham tDCS (*p* = 0.16; *p* = 0.11), or between active rTMS and active tDCS (*p* = 0.89; *p* = 0.69). Of the remaining exploratory comparisons, only two had unadjusted *p* values below 0.05 (WHOQOL-BREF at 1 week, active vs. sham rTMS; paresthesia/dysesthesia at 4 weeks, sham rTMS vs. sham tDCS), which are likely chance findings given the number of comparisons.

### 3.3. Patient-Reported Treatment Preferences

Patient-reported treatment preference responses were assessed, and the results are presented in Figure 3.

In the active group (*n* = 12), 66.7% of participants rated rTMS as more helpful, 16.7% rated tDCS as more helpful, and 16.7% reported no difference. In the sham group (*n* = 13), 23.1% rated rTMS as more helpful, and 76.9% reported no difference. The distribution of responses differed between the active and sham groups (*p* = 0.005). Because participants were aware of which modality they had received and blinding was not formally assessed, this exploratory finding should be interpreted with caution.

### 3.4. Safety and Tolerability

Adverse events are summarized in Table 4.

No serious adverse events were observed in any of the stimulation conditions. Mild adverse events were reported in a small number of patients, including headache, dizziness, and transient increases in pain intensity.

In the rTMS group, headache (7.7%), dizziness (7.7%), and transient pain exacerbation (15.4%) were reported. In the tDCS group, headache (7.7%) and transient pain exacerbation (7.7%) were observed. Similar mild adverse events were also reported in the sham condition.

Adverse events were infrequent and descriptively similar across stimulation conditions. Given the small number of events, no formal between-condition hypothesis testing was performed.

## 4. Discussion

In this exploratory randomized, sham-controlled crossover trial in patients with CNP, neither active rTMS nor active tDCS over M1 produced a statistically significant reduction in pain intensity compared with modality-matched sham stimulation, and no difference between the two active modalities was demonstrated.

NIBS, including rTMS and tDCS, modulates cortical excitability and neuroplasticity through distinct mechanisms. rTMS generates electric fields capable of inducing neuronal activation, whereas tDCS primarily modulates neuronal membrane potentials in a subthreshold manner [27].

At 1 week, the estimated difference between active and sham rTMS was 0.76 NRS points in the unadjusted factorial model, but its 95% confidence interval included zero (−0.005 to 1.53), and the estimate was attenuated to 0.42 points (95% CI −0.37 to 1.21) after adjustment for the baseline age imbalance. Because age was associated with greater pain reduction and the active group was older, the unadjusted treatment estimate may have been influenced by the baseline age imbalance. Active tDCS did not differ from sham tDCS. Active rTMS also did not differ significantly from active tDCS (age-adjusted difference 0.65 points, 95% CI −0.09 to 1.40), and the condition × modality interaction was not significant; thus, the present data provide no statistical evidence that rTMS is superior to tDCS, and the absence of a detectable tDCS effect cannot be taken as evidence of rTMS superiority. All estimates were smaller than the 1.5-point difference assumed in the sample size calculation, and with fewer participants than planned, the study had limited power to detect modest effects of either modality.

The M1 was selected as the stimulation target because of its established role in pain modulation and because it has the strongest level of evidence among cortical targets for neuropathic pain [6]. Previous sham-controlled studies have reported short-term analgesic effects of M1-rTMS in CNP conditions, including post-stroke pain and spinal cord injury, which are thought to involve activation of descending inhibitory pathways [28]. The point estimates for rTMS in the present study were in the same direction; however, because they were not statistically significant and were sensitive to age adjustment, they should be regarded as hypothesis-generating rather than as confirmation of these previous findings.

The responder analysis provides further context for the mean NRS findings. At 1 week, 3 of 13 participants (23.1%) had a pain reduction of at least 30% after active rTMS and 2 (15.4%) had a reduction of at least 50%, whereas no participant reached either threshold after sham rTMS. However, none of the active-versus-sham rTMS responder comparisons was statistically significant (≥30% reduction, *p* = 0.22; ≥50% reduction, *p* = 0.48; ≥2-point reduction, *p* = 0.38), and the confidence intervals were wide (Table 3). In addition, although thresholds for individual improvement cannot be applied directly to differences between group means, even the upper confidence limit of the unadjusted 1-week estimate (1.53 points) was below the 2-point change commonly regarded as clinically meaningful for individual patients [25,26]. These findings therefore do not establish a clinically meaningful benefit of short-course rTMS; whether a subset of patients responds would need to be tested in larger trials.

No consistent differences were observed in secondary outcomes, including neuropathic pain characteristics, mood, sleep quality, and quality of life. Two isolated comparisons had unadjusted *p* values below 0.05, but given the number of exploratory comparisons, these are most likely chance findings. Because the primary outcome itself did not differ significantly from sham, the absence of effects on secondary outcomes should not be interpreted as a dissociation between pain intensity and broader outcomes; rather, the study lacked the power to detect modest changes in these domains. Of note, in a multicentre trial of M1-rTMS for neuropathic pain, pain intensity improved, whereas mood, sleep, quality of pain, and quality of life did not differ from sham stimulation [29], and short-course M1 stimulation would not be expected to reproduce the antidepressant effects of prefrontal rTMS delivered over several weeks [6,30].

At 4 weeks, no comparison between active and modality-matched sham stimulation was statistically significant. Although the mean NRS reduction after active rTMS at 4 weeks was similar to that at 1 week, reductions were also observed after sham stimulation, and the age-adjusted difference between active and sham rTMS was close to zero (0.13 points, 95% CI −1.14 to 1.39). The present data therefore provide no evidence of a sustained effect. Whether repeated or maintenance stimulation could produce larger or more durable effects remains to be determined.

We also found no evidence of an analgesic effect of tDCS, although the study was not large enough to exclude a modest effect. The stimulation parameters were based on commonly used clinical protocols, and five sessions may be insufficient given the cumulative and subthreshold nature of tDCS-induced neuromodulation [31].

Small improvements were observed after sham stimulation, particularly after sham rTMS, which may reflect expectancy and procedure-related placebo effects [32]. Because active rTMS did not differ significantly from modality-matched sham, whether active rTMS provides benefit beyond placebo could not be established in this sample. This question is further complicated by the subjective nature of pain ratings and the absence of a formal assessment of blinding.

Patient-reported treatment preference favoured rTMS more often in the active group than in the sham group. However, this was an exploratory outcome; participants knew which modality they had received, and the preference was not accompanied by a significant difference in the primary outcome; it should therefore not be taken as evidence of efficacy.

Overall, the present findings do not establish the efficacy of short-course M1-rTMS or tDCS for CNP. Rather, they provide effect-size estimates and feasibility data that may inform the design of adequately powered trials.

This study has several limitations. First, the number of participants enrolled was smaller than originally planned, resulting in a relatively small sample size. This limited the statistical power to detect modest between-group differences and reduced the precision of the estimated treatment effects. Because none of the primary comparisons reached statistical significance, the present results should be regarded as inconclusive rather than as evidence of no effect, and a larger, adequately powered study is needed. The single-centre design may also limit the generalisability of the results. In addition, the active group was older than the sham group, and age was associated with greater pain reduction; after adjustment for age, the estimated effect of rTMS was substantially attenuated. Residual confounding by age or by other characteristics that were not balanced in this small sample cannot be excluded.

Second, blinding was not complete. Participants did not know whether they received active or sham stimulation. However, they could tell whether they received rTMS or tDCS, because the two procedures look and feel very different. Since pain is a subjective outcome, what participants expected from each procedure may have affected their pain ratings. Our data suggest that this may have happened to some degree: pain decreased slightly more after sham rTMS than after sham tDCS, although this difference was not significant. Because the sham group also received both procedures, these expectations should have affected the active and sham groups in a similar way. We also did not formally check whether participants could differentiate active from sham stimulation. This is possible because the sham rTMS coil did not produce the same scalp sensation as active rTMS, and active tDCS at 2 mA may cause more skin sensation than sham tDCS. Finally, the investigator who delivered the stimulation knew which treatment each participant received. Future trials should formally assess whether blinding was successful.

Third, stimulation targeting was performed without MRI-guided neuronavigation. Although motor hotspot-based targeting is a commonly used and clinically feasible approach for M1 stimulation, neuronavigation may improve anatomical targeting precision and reproducibility across participants [33]. Future studies incorporating neuronavigation may therefore allow more consistent stimulation of the intended cortical target.

Fourth, outcomes were assessed only at 1 and 4 weeks after stimulation, without intermediate measurements; the time course of any response could therefore not be characterised. More frequent assessments would help clarify the onset and duration of any analgesic effect.

Fifth, carry-over effects are a concern in any crossover trial. In our study, the 4-week assessment after the first treatment was also used as the baseline for the second treatment. We therefore checked whether pain had returned to baseline before the second treatment began.

In patients who received active rTMS first, mean pain before the second treatment was close to the initial level (6.57 ± 1.90 vs. 6.86 ± 0.90). However, five of seven patients still had pain scores at least 1 point lower than at the start (Supplementary Table S2). Some residual effect of rTMS in individual patients is therefore possible. If present, it would most likely have reduced the apparent effect of tDCS given in the second period.

Several findings suggest that this did not have a large effect on our results. First, tDCS given after rTMS showed a similar change in pain to tDCS given first (0.33 ± 1.03 vs. 0.50 ± 1.52). Second, the period effect was very small (−0.09; 95% CI −0.61 to 0.44 in the age-adjusted model). Third, when we analysed only the first treatment period, where carry-over cannot occur, the 1-week difference between active rTMS and sham rTMS was similar in direction (0.81; 95% CI −0.34 to 1.96; Supplementary Table S3).

Even so, these checks have limited power in a small sample. The confidence interval of the carry-over test was wide (−2.30 to 2.64 points), so a clinically meaningful carry-over effect cannot be ruled out. Future studies should use a longer washout period and confirm that pain has returned to baseline before crossover.

Sixth, the study population was also heterogeneous. Patients had different causes of CNP, including stroke, demyelinating disease, and spinal cord lesions, and these conditions may respond differently to neuromodulation. The proportion of each cause also differed between the active and sham groups. Future trials should include more homogeneous populations or be large enough to compare different causes.

Finally, the study relied primarily on patient-reported outcomes without incorporating objective neurophysiological or imaging biomarkers. Future studies combining clinical outcomes with objective measures may help clarify the neurophysiological mechanisms underlying any analgesic effects of NIBS.

In this exploratory randomized, sham-controlled crossover trial in patients with CNP, neither high-frequency rTMS nor anodal tDCS over M1 showed statistically significant superiority over modality-matched sham stimulation at 1 or 4 weeks, and no difference between the two modalities was demonstrated. The point estimate for rTMS at 1 week was directionally favourable but imprecise and was attenuated after adjustment for a baseline age imbalance. Both modalities were well tolerated.

These findings do not establish the efficacy of either modality for CNP. They may, however, inform the design of adequately powered trials with balanced baseline characteristics, formal assessment of blinding, and longer follow-up to determine whether M1-rTMS provides clinically meaningful benefit and which patients, if any, are most likely to respond.

## Figures and Tables

**Figure 1:**
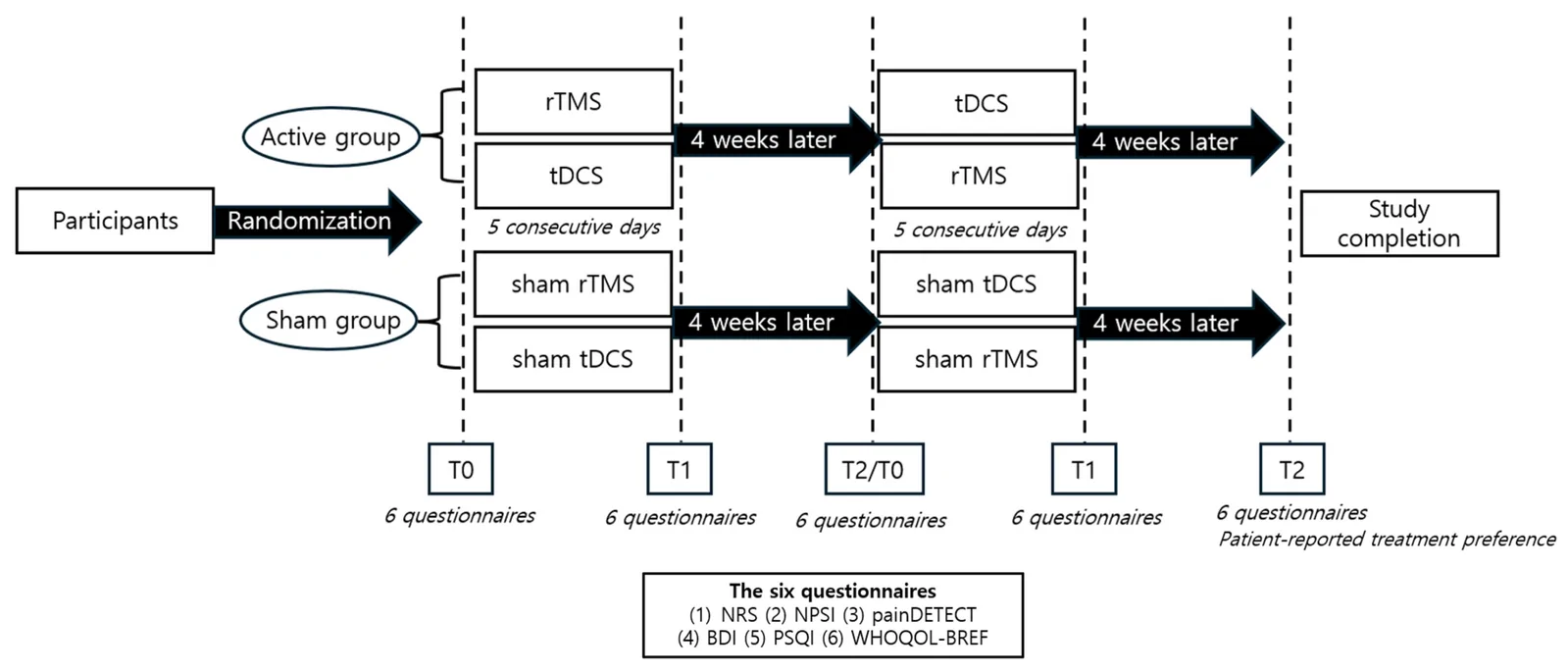
Study design and crossover protocol.

**Figure 2:**
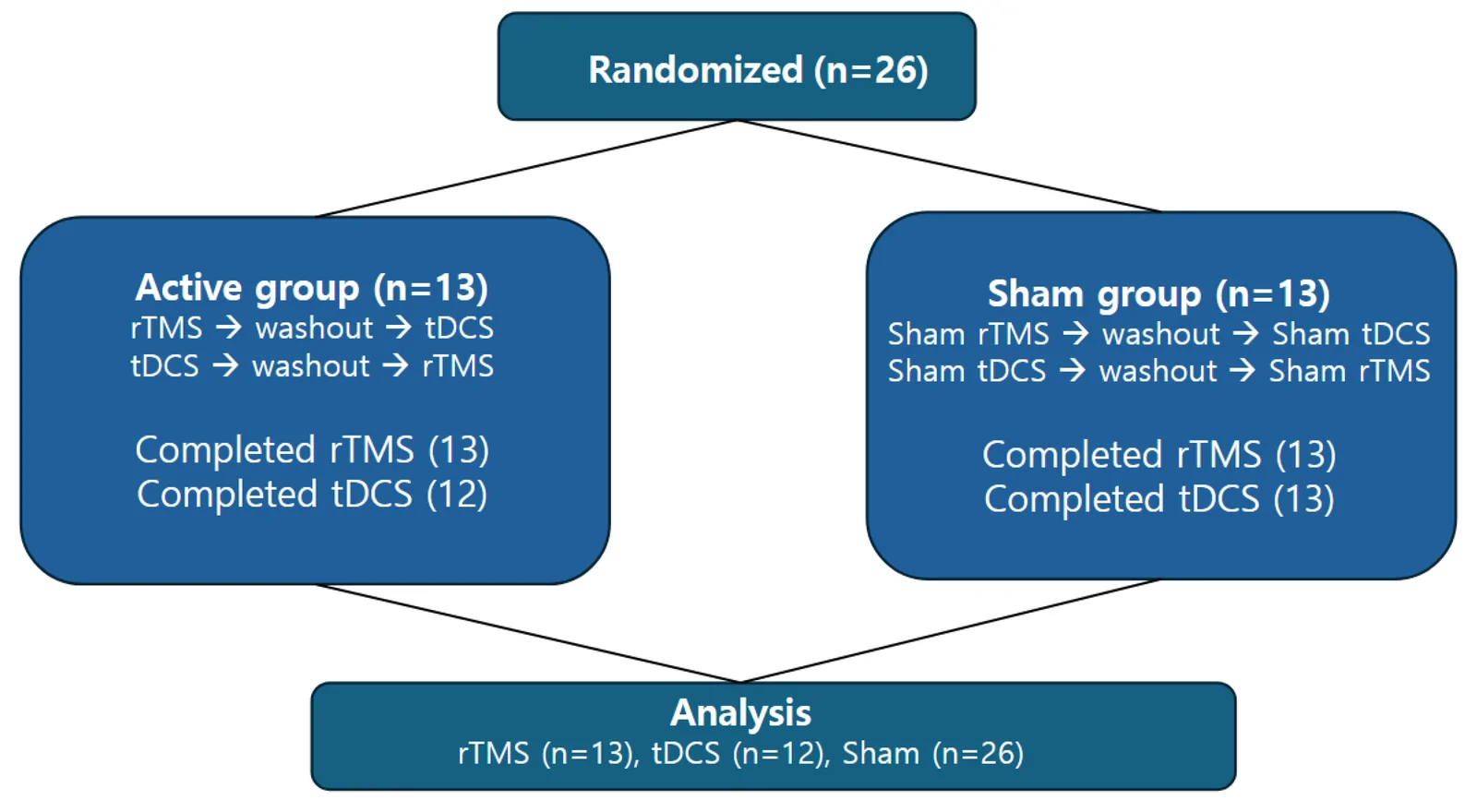
CONSORT [11] flow diagram of the study.

**Figure 3:**
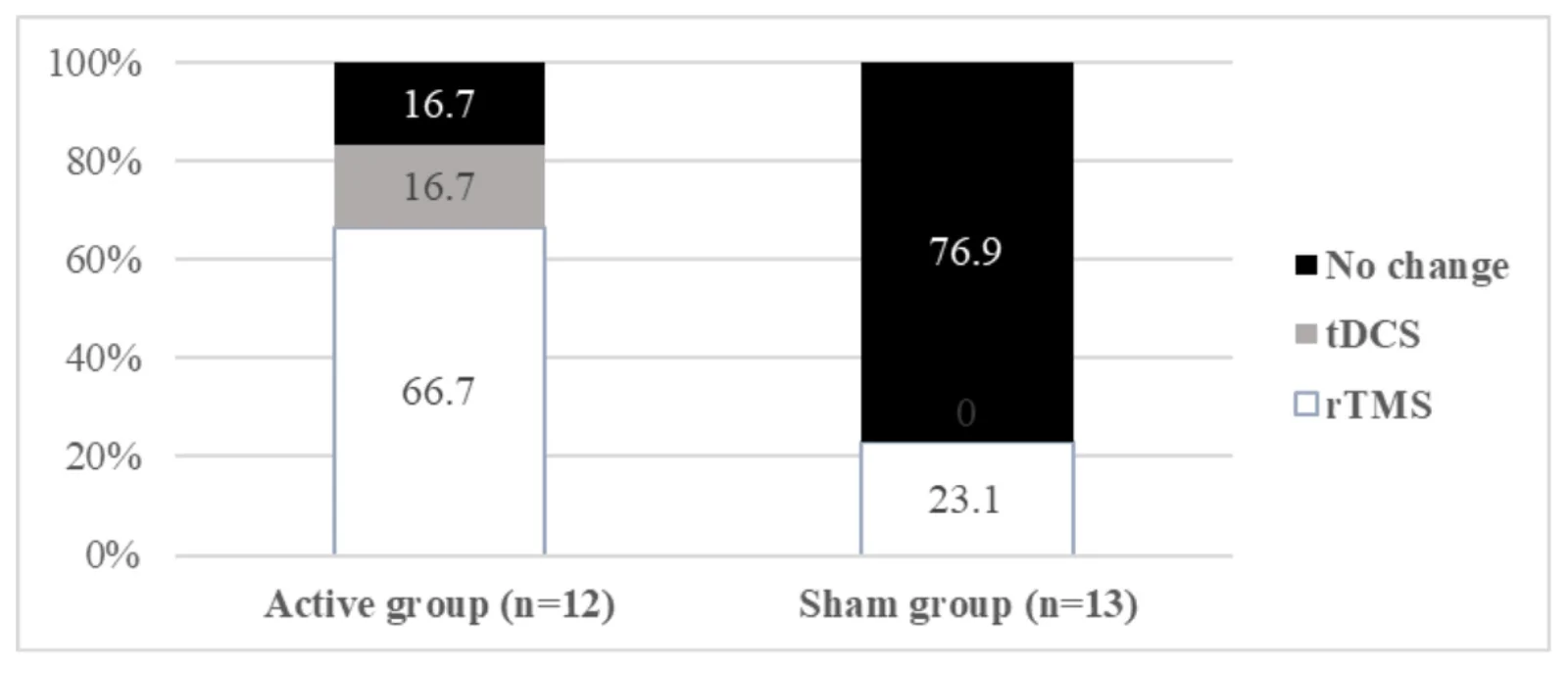
Distribution of patient-reported treatment preference responses according to stimulation modality. Stacked bar charts showing the distribution of patient-reported treatment preference responses following each stimulation modality in the active (*n* = 12) and sham (*n* = 13) groups. A higher proportion of participants in the active group than in the sham group rated rTMS as more helpful. The distribution of responses differed significantly between groups (*p* = 0.005). rTMS, repetitive transcranial magnetic stimulation; tDCS, transcranial direct current stimulation.

**Table 1:** Numerical Rating Scale (NRS) pain scores and changes from the start of each treatment period, by stimulation condition and modality.

	Active rTMS (*n* = 13)	Sham rTMS (*n* = 13)	Active tDCS (*n* = 12)	Sham tDCS (*n* = 13)
**T0**	6.4 ± 1.8	6.2 ± 2.8	6.3 ± 1.9	6.5 ± 2.4
**T1 (1 week)**	5.3 ± 2.1	5.9 ± 2.7	5.8 ± 1.6	6.5 ± 2.4
**T2 (4 weeks)**	5.3 ± 2.6	5.9 ± 2.6	6.1 ± 2.2	5.8 ± 2.5
**ΔNRS T0–T1**	1.1 ± 1.3	0.3 ± 0.8	0.4 ± 1.2	0.0 ± 0.4
**ΔNRS T0–T2**	1.1 ± 2.3	0.3 ± 1.3	0.2 ± 1.6	0.6 ± 1.2

Values are presented as mean ± standard deviation. Each column corresponds to one cell of the 2 × 2 factorial design. Active rTMS and active tDCS observations were contributed by participants in the active group (*n* = 13; one participant did not complete the tDCS period), and sham rTMS and sham tDCS observations were contributed by participants in the sham group (*n* = 13); within each group, the two modalities were received by the same participants in a crossover sequence. T0, start of each treatment period; T1, immediately after completion of the five-session treatment course (approximately 1 week after T0); T2, 4 weeks after treatment. For Period 2, T0 corresponded to the 4-week assessment of Period 1. ΔNRS was calculated as T0 minus the corresponding follow-up score, so positive values indicate pain reduction. Inferential comparisons are presented in Table 2. NRS, Numerical Rating Scale; rTMS, repetitive transcranial magnetic stimulation; tDCS, transcranial direct current stimulation.

**Table 2:** Factorial linear mixed-effects models for change in NRS pain intensity at 1 and 4 weeks, unadjusted and adjusted for age (26 participants, 51 observations). (**A**) Unadjusted model. (**B**) Age-adjusted model.

	ΔNRS T0–T1 (1 Week)			ΔNRS T0–T2 (4 Weeks)		
Effect	Estimate (SE)	95% CI	*p*	Estimate (SE)	95% CI	*p*
***(A) Unadjusted model***						
*Fixed effects*						
Intercept	0.36 (0.35)	−0.32 to 1.04	0.301	−0.14 (0.57)	−1.27 to 0.98	0.806
Condition (active vs. sham) ^a^	0.76 (0.39)	−0.005 to 1.53	0.051	0.83 (0.65)	−0.44 to 2.10	0.198
Modality (tDCS vs. rTMS) ^b^	−0.31 (0.38)	−1.06 to 0.43	0.406	0.33 (0.60)	−0.85 to 1.52	0.579
Condition × modality ^c^	0.35 (0.54)	−0.72 to 1.41	0.522	1.30 (0.86)	−0.40 to 2.99	0.134
Period (second vs. first)	−0.09 (0.27)	−0.62 to 0.44	0.739	0.35 (0.43)	−0.49 to 1.20	0.415
Sequence (tDCS first vs. rTMS first)	0.00 (0.29)	−0.57 to 0.56	0.987	0.48 (0.49)	−0.48 to 1.44	0.327
*Model-derived contrasts*						
Active rTMS vs. sham rTMS	0.76 (0.39)	−0.005 to 1.53	0.051	0.83 (0.65)	−0.44 to 2.10	0.198
Active tDCS vs. sham tDCS	0.41 (0.40)	−0.37 to 1.20	0.299	−0.46 (0.66)	−1.75 to 0.83	0.484
Active rTMS vs. active tDCS	0.66 (0.39)	−0.10 to 1.42	0.087	0.96 (0.62)	−0.25 to 2.17	0.120
Sham rTMS vs. sham tDCS	0.31 (0.38)	−0.43 to 1.06	0.406	−0.33 (0.60)	−1.52 to 0.85	0.579
*Random effects (variance)*						
Participant (random intercept)	0.06			0.35		
Residual	0.93			2.36		
***(B) Age-adjusted model***						
*Fixed effects*						
Intercept	0.64 (0.35)	−0.06 to 1.33	0.071	0.44 (0.57)	−0.67 to 1.55	0.439
Condition (active vs. sham) ^a^	0.42 (0.40)	−0.37 to 1.21	0.297	0.13 (0.65)	−1.14 to 1.39	0.844
Modality (tDCS vs. rTMS) ^b^	−0.31 (0.37)	−1.05 to 0.42	0.400	0.34 (0.60)	−0.84 to 1.51	0.574
Condition × modality ^c^	0.34 (0.53)	−0.71 to 1.39	0.526	1.27 (0.86)	−0.40 to 2.95	0.137
Period (second vs. first)	−0.09 (0.27)	−0.61 to 0.44	0.747	0.36 (0.43)	−0.47 to 1.20	0.395
Sequence (tDCS first vs. rTMS first)	−0.23 (0.29)	−0.79 to 0.33	0.416	0.01 (0.46)	−0.89 to 0.90	0.986
Age (per year, centred)	0.04 (0.02)	0.01 to 0.07	0.023	0.08 (0.03)	0.03 to 0.13	0.003
*Model-derived contrasts*						
Active rTMS vs. sham rTMS	0.42 (0.40)	−0.37 to 1.21	0.297	0.13 (0.65)	−1.14 to 1.39	0.844
Active tDCS vs. sham tDCS	0.08 (0.41)	−0.72 to 0.88	0.843	−1.14 (0.66)	−2.43 to 0.14	0.081
Active rTMS vs. active tDCS	0.65 (0.38)	−0.09 to 1.40	0.087	0.94 (0.61)	−0.26 to 2.13	0.125
Sham rTMS vs. sham tDCS	0.31 (0.37)	−0.42 to 1.05	0.400	−0.34 (0.60)	−1.51 to 0.84	0.574
*Random effects (variance)*						
Participant (random intercept)	0.00			0.00		
Residual	0.91			2.32		

ΔNRS is the NRS at the start of each treatment period minus the NRS at follow-up; positive values indicate greater pain reduction. Models included condition (active vs. sham), modality (rTMS vs. tDCS), the condition × modality interaction, treatment period, and treatment sequence as fixed effects, with age (centred at the sample mean) as an additional covariate in the age-adjusted model (**B**), and a random intercept for participant. Models were fitted separately for the 1-week and 4-week outcomes by restricted maximum likelihood. Reference categories were sham, rTMS, the first period, and the rTMS-first sequence. ^a^ Difference between active and sham stimulation within rTMS (equivalent to the active rTMS vs. sham rTMS contrast). ^b^ Difference between tDCS and rTMS within sham stimulation. ^c^ Difference in the active-versus-sham effect between modalities (active rTMS − sham rTMS) − (active tDCS − sham tDCS); positive values indicate a larger active-versus-sham effect for rTMS. Model-derived contrasts are expressed as the first condition minus the second; positive values favour the first condition. In the age-adjusted models, the participant random-intercept variance was estimated at zero (boundary estimate). Active rTMS and active tDCS conditions include 13 and 12 observations, respectively; sham rTMS and sham tDCS conditions include 13 observations each. SE, standard error; CI, confidence interval; NRS, Numerical Rating Scale; rTMS, repetitive transcranial magnetic stimulation; tDCS, transcranial direct current stimulation.

**Table 3:** Responder analysis of pain intensity (NRS) after active and sham stimulation.

	Active rTMS vs. Sham rTMS				Active tDCS vs. Sham tDCS			
Outcome	Active rTMS (*n* = 13)	Sham rTMS (*n* = 13)	Difference (95% CI)	*p*	Active tDCS (*n* = 12)	Sham tDCS (*n* = 13)	Difference (95% CI)	*p*
***1 week (T1)***								
Pain reduction, %, mean ± SD	17.5 ± 21.9	3.8 ± 9.4	13.7 (−0.3 to 27.7)	0.055	2.5 ± 27.1	−0.1 ± 5.5	2.7 (−14.7 to 20.0)	0.74
≥30% reduction, *n*/*N* (%)	3/13 (23.1)	0/13 (0.0)	23.1 (−4.2 to 50.3)	0.22	0/12 (0.0)	0/13 (0.0)	0.0 (−22.8 to 24.3)	1.00
≥50% reduction, *n*/*N* (%)	2/13 (15.4)	0/13 (0.0)	15.4 (−10.0 to 42.2)	0.48	0/12 (0.0)	0/13 (0.0)	0.0 (−22.8 to 24.3)	1.00
≥2-point reduction, *n*/*N* (%)	5/13 (38.5)	2/13 (15.4)	23.1 (−10.9 to 51.3)	0.38	3/12 (25.0)	0/13 (0.0)	25.0 (−2.9 to 53.2)	0.096
***4 weeks (T2)***								
Pain reduction, %, mean ± SD	16.1 ± 37.5	4.6 ± 24.4	11.5 (−14.3 to 37.4)	0.36	−0.4 ± 32.0	11.1 ± 19.3	−11.5 (−33.9 to 11.0)	0.30
≥30% reduction, *n*/*N* (%)	4/13 (30.8)	1/13 (7.7)	23.1 (−8.3 to 50.7)	0.32	1/12 (8.3)	2/13 (15.4)	−7.1 (−34.8 to 22.2)	1.00
≥50% reduction, *n*/*N* (%)	3/13 (23.1)	1/13 (7.7)	15.4 (−14.3 to 43.3)	0.59	0/12 (0.0)	2/13 (15.4)	−15.4 (−42.2 to 11.3)	0.48
≥2-point reduction, *n*/*N* (%)	6/13 (46.2)	2/13 (15.4)	30.8 (−4.6 to 57.8)	0.20	3/12 (25.0)	1/13 (7.7)	17.3 (−13.0 to 46.2)	0.32

Percentage pain reduction was calculated for each participant as (NRS at the start of the treatment period − NRS at follow-up)/NRS at the start of the treatment period × 100; positive values indicate pain reduction. Each active condition was compared with its matching sham condition. Because participants in the active and sham groups are different people, these comparisons involve independent participants. Differences are expressed as active minus sham (percentage points for proportions; percentage for mean pain reduction). Confidence intervals for differences in proportions were calculated using the Newcombe hybrid score method; *p* values are from Fisher’s exact test for proportions and Welch’s *t*-test for mean percentage reduction. The ≥30% and ≥50% thresholds correspond to moderately and substantially important improvement and a ≥2-point reduction to clinically meaningful improvement in chronic pain [25,26]. Given the small sample, these analyses have limited statistical power and should be interpreted as exploratory. NRS, Numerical Rating Scale; rTMS, repetitive transcranial magnetic stimulation; tDCS, transcranial direct current stimulation; CI, confidence interval.

**Table 4:** Comparison of side effects and safety profile reported after stimulation sessions by the rTMS, tDCS, and sham groups.

	Active rTMS Group (*n* = 13)	Active tDCS Group (*n* = 13)	Sham Group (13 Participants, 26 Observations)
Headache, *n* (%)	1 (7.7%)	1 (7.7%)	1 (3.8%)
Vertigo, *n* (%)	1 (7.7%)		1 (3.8%)
Temporary increase in pain intensity, *n* (%)	2 (15.4%)	1 (7.7%)	1 (3.8%)

rTMS, repetitive transcranial magnetic stimulation; tDCS, transcranial direct current stimulation.

## Data Availability

The data presented in this study are available upon request from the corresponding author due to ongoing related research and data management restrictions.

## Supplementary Materials

The following supporting information can be downloaded at: https://www.mdpi.com/article/10.3390/brainsci16101078/s1, Table S1. Demographic data and baseline patient-reported outcome measures; Table S2. Pain scores (NRS) at the start of each treatment period, by treatment sequence; Table S3. Change in pain intensity by stimulation condition and treatment period, and modality-matched comparisons using first-period data only; Table S4. Exploratory comparisons of change scores in secondary outcomes according to stimulation condition and modality.

## Abbreviations

The following abbreviations are used in this manuscript: CNP, central neuropathic pain; NIBS, non-invasive brain stimulation; rTMS, repetitive transcranial magnetic stimulation; tDCS, transcranial direct current stimulation; M1, primary motor cortex; vRMT, visual resting motor threshold; NRS, Numerical Rating Scale; NPSI, Neuropathic Pain Symptom Inventory; BDI, Beck Depression Inventory; PSQI, Pittsburgh Sleep Quality Index; WHOQOL-BREF, World Health Organization Quality of Life-BREF; PGIC, Patient Global Impression of Change.
