# Supplementary material for: Non-Invasive Brain Stimulation for Central Neuropathic Pain: An Exploratory Randomized Sham-Controlled Crossover Trial
Source: Brain Sci. 2026 Oct 8;16(10):1078. doi: 10.3390/brainsci16101078 (PMC13702139; doi:10.3390/brainsci16101078)
Supplement: Supplementary file 1 [file brainsci-16-01078-s001.zip › brainsci-4582727-supplementary.pdf]

Supplementary Table S1. Demographic data and baseline patient-reported outcome measures.

	Active group(n=13)	Sham group(n=13)	*p* value
Age (years, mean±SD)	64.8±6.9	56.2±10.4	0.04*
Sex, Male, n (%)	8 (61.5%)	9 (69.2%)	1.0
Final diagnosis, n (%)			0.3
Transverse myelitis or CNS demyelinating disease	7 (53.8%)	3 (23.1%)	
Spinal cord infarction	0 (0.0%)	1 (7.7%)	
Spinal cord tumor	0 (0.0%)	1 (7.7%)	
Cerebral infarction	6 (46.2%)	7 (53.8%)	
Other (cerebellar ataxia)	0 (0.0%)	1 (7.7%)	
Disease duration (months, mean±SD)	105.0±94.1	85.3±51.0	0.7
Stimulation site, Right, n (%)	3 (23.1%)	7 (53.8%)	0.2
Medical treatment			
Number of drugs (mean±SD)	2.5±1.0	2.2±1.1	0.4
Antiepileptics, n (%)	12 (92.3%)	12 (92.3%)	1.0
Antidepressant, n (%)	7 (53.8%)	5 (38.5%)	0.7
Tramadol, n (%)	6 (46.2%)	5 (38.5%)	1.0
Strong opioid, n (%)	2 (15.4%)	0 (0.0%)	0.5
Amount of drugs			
Pregabalin (mg, mean±SD)	230.6±110.9 (n=9)	265.0±147.8 (n=10)	0.6
Gabapentin (mg, mean±SD)	900.0±519.6 (n=3)	1083.3±534.5 (n=6)	0.6
TCA (mg, mean±SD)	11.0±5.5 (n=5)	6.7±2.9 (n=3)	0.3
Duloxetine (mg, mean±SD)	67.5±37.7 (n=4)	60.0±52.0 (n=3)	0.8
Tramadol (mg, mean±SD)	112.5±37.5 (n=5)	139.3±51.8 (n=7)	0.3
NRS (0-10), (mean±SD)	6.5±1.4	6.6±2.6	0.6
NPSI total (0-110), (mean±SD)	38.9±24.6	42.8±17.9	0.4
time score (0-10)	6.5±2.4	5.9±2.4	0.7
pain quality (0-100)	32.5±22.5	36.9±17.2	0.4
superficial pain (0-10)	3.0±3.1	2.5±3.0	0.7
deep pain (0-10)	3.6±3.0	3.2±3.5	0.7
paroxysmal pain (0-10)	2.7±3.2	2.7±2.9	0.7
evoked pain (0-10)	2.5±2.8	4.2±3.0	0.1
dysesthesia (0-10)	4.8±1.8	5.1±2.6	0.7
painDETECT (0-35), (mean±SD)	13.9±7.7	16.4±6.9	0.4
BDI (0-63), (mean±SD)	24.7±14.7	19.5±9.6	0.7
PSQI (0-21), (mean±SD)	12.5±6.8	10.1±5.8	0.4
WHOQOL-BREF (0-130), (mean±SD)	63.8±21.3	66.2±14.4	0.7

SD, standard deviation; CNS, central nervous system; TCA, tricyclic antidepressants; NRS, Numeric Rating Scale; NPSI, Neuropathic Pain Symptom Inventory; BDI, Beck Depression Inventory; PSQI, Pittsburgh Sleep Quality Index, WHOQOL-BREF, World Health Organization Quality of Life-brief version

* indicates statistical significance at $p < 0.05$.

Supplementary Table S2. Pain scores (NRS) at the start of each treatment period, by treatment sequence.

Sequence	n	NRS at start of period 1	NRS at start of period 2	Difference (95% CI)	Median difference	≥1 point lower, n/N	p
Active rTMS → active tDCS	7	6.86 ± 0.90	6.57 ± 1.90	0.29 (−1.69 to 2.26)	1.0	5/7	0.74
Active tDCS → active rTMS	6	6.00 ± 1.79	5.83 ± 2.40	0.17 (−2.08 to 2.41)	1.0	4/6	0.86
Sham rTMS → sham tDCS	6	7.17 ± 1.72	6.83 ± 0.98	0.33 (−1.62 to 2.29)	0.5	3/6	0.68
Sham tDCS → sham rTMS	7	6.14 ± 3.18	5.43 ± 3.36	0.71 (−0.67 to 2.10)	0.0	2/7	0.25

Values are mean ± standard deviation unless otherwise stated. The NRS at the start of period 2 is the 4-week assessment of period 1, obtained after the 4-week washout. Difference = NRS at the start of period 1 minus NRS at the start of period 2; positive values indicate that pain had not fully returned to the initial level. "≥1 point lower" indicates the number of participants whose NRS at the start of period 2 was at least 1 point lower than at the start of period 1. p values are from paired t-tests. NRS, Numerical Rating Scale; rTMS, repetitive transcranial magnetic stimulation; tDCS, transcranial direct current stimulation; CI, confidence interval.

Supplementary Table S3. Change in pain intensity by stimulation condition and treatment period, and modality-matched comparisons using first-period data only.

(A) Change in NRS by condition and treatment period

	Period 1			Period 2		
**Condition**	**n**	**ΔNRS T0–T1**	**ΔNRS T0–T2**	**n**	**ΔNRS T0–T1**	**ΔNRS T0–T2**
Active rTMS	7	1.14 ± 1.07	0.29 ± 2.14	6	1.00 ± 1.55	2.00 ± 2.19
Sham rTMS	6	0.33 ± 0.82	0.33 ± 1.86	7	0.29 ± 0.76	0.29 ± 0.49
Active tDCS	6	0.50 ± 1.52	0.17 ± 2.14	6	0.33 ± 1.03	0.17 ± 1.17
Sham tDCS	7	0.00 ± 0.00	0.71 ± 1.50	6	0.00 ± 0.63	0.50 ± 0.84

(B) Between-condition comparisons, first period only

	ΔNRS T0–T1			ΔNRS T0–T2		
**Comparison**	**Difference (95% CI)**	***p* (Welch)**	***p* (Mann–Whitney)**	**Difference (95% CI)**	***p* (Welch)**	***p* (Mann–Whitney)**
Active rTMS vs. sham rTMS	0.81 (−0.34 to 1.96)	0.151	0.117	−0.05 (−2.49 to 2.39)	0.967	1.000
Active tDCS vs. sham tDCS	0.50 (−1.09 to 2.09)	0.456	0.256	−0.55 (−2.91 to 1.81)	0.611	0.824
Active rTMS vs. active tDCS	0.64 (−1.03 to 2.32)	0.408	0.554	0.12 (−2.51 to 2.75)	0.922	1.000
Sham rTMS vs. sham tDCS	0.33 (−0.52 to 1.19)	0.363	0.355	−0.38 (−2.50 to 1.74)	0.696	0.876

Values in (A) are mean ± standard deviation. ΔNRS is calculated as the NRS at the start of each period minus the NRS at follow-up, so positive values indicate pain reduction. T0, start of the treatment period; T1, immediately after the five-session course (approximately 1 week); T2, 4 weeks after treatment. In (B), only data from the first treatment period were used (active rTMS, n = 7; sham rTMS, n = 6; active tDCS, n = 6; sham tDCS, n = 7), so carry-over from a previous treatment cannot affect these estimates. Because each participant contributed only one first-period observation, all comparisons in (B) involve independent participants. Differences are expressed as the first condition minus the second; positive values favour the first condition. Confidence intervals and p values are from Welch's t-test; p values from the Mann–Whitney U test are also shown. Given the small number of participants per condition, these analyses have limited statistical power and are presented as sensitivity analyses. NRS, Numerical Rating Scale; rTMS, repetitive transcranial magnetic stimulation; tDCS, transcranial direct current stimulation; CI, confidence interval.

Supplementary Table S4. Exploratory comparisons of change scores in secondary outcomes according to stimulation condition and modality.

Outcome	Change score, median (IQR)				*p value*			
	Active rTMS (n = 13)	Sham rTMS (n = 13)	Active tDCS (n = 12)	Sham tDCS (n = 13)	Active vs. sham rTMS [a]	Active vs. sham tDCS [a]	Active rTMS vs. active tDCS [b]	Sham rTMS vs. sham tDCS [b]
1 week (T0–T1)								
NPSI total	0.0 (0.0 to 2.0)	0.0 (0.0 to 0.0)	1.0 (0.0 to 3.2)	0.0 (0.0 to 0.0)	0.91	0.16	0.89	0.48
Superficial pain	0.0 (0.0 to 0.0)	0.0 (0.0 to 0.0)	0.0 (0.0 to 1.0)	0.0 (0.0 to 0.0)	0.64	0.16	0.75	0.75
Deep pain	0.0 (0.0 to 0.0)	0.0 (0.0 to 0.0)	0.0 (−0.5 to 0.6)	0.0 (0.0 to 0.0)	0.77	0.93	0.63	0.88
Paroxysmal pain	0.0 (0.0 to 0.0)	0.0 (0.0 to 0.0)	0.0 (−0.2 to 0.0)	0.0 (0.0 to 0.0)	1.00	0.68	0.25	0.50
Evoked pain	0.0 (0.0 to 0.0)	0.0 (0.0 to 0.0)	0.0 (0.0 to 0.0)	0.0 (0.0 to 0.0)	0.47	0.71	0.56	0.12
Paresthesia/dysesthesia	0.0 (0.0 to 1.0)	0.0 (0.0 to 0.0)	0.0 (0.0 to 0.9)	0.0 (0.0 to 0.0)	0.62	0.28	0.92	0.16
painDETECT	0.0 (0.0 to 0.0)	0.0 (0.0 to 0.0)	0.0 (−2.2 to 0.8)	0.0 (0.0 to 0.0)	0.36	0.86	1.00	0.50
BDI	0.0 (0.0 to 3.0)	0.0 (0.0 to 0.0)	0.0 (−0.2 to 1.8)	0.0 (0.0 to 1.0)	0.25	0.98	0.63	1.00
PSQI	0.0 (0.0 to 0.0)	0.0 (0.0 to 0.0)	0.0 (0.0 to 1.0)	0.0 (0.0 to 0.0)	1.00	0.63	0.69	0.75
WHOQOL-BREF	1.0 (0.0 to 2.0)	0.0 (0.0 to 0.0)	0.0 (−0.2 to 0.2)	0.0 (−1.0 to 2.0)	0.02	0.80	0.07	0.77
4 weeks (T0–T2)								
NPSI total	1.0 (0.0 to 2.0)	0.0 (−1.0 to 4.0)	3.0 (0.5 to 8.5)	0.0 (−4.0 to 2.0)	0.80	0.11	0.69	0.11
Superficial pain	0.0 (0.0 to 0.0)	0.0 (0.0 to 0.0)	0.5 (0.0 to 1.0)	0.0 (−1.0 to 0.0)	0.88	0.09	1.00	0.23
Deep pain	0.0 (0.0 to 0.0)	0.0 (0.0 to 0.0)	0.2 (−1.2 to 0.6)	0.0 (−0.5 to 0.0)	0.67	0.43	0.52	0.60
Paroxysmal pain	0.0 (0.0 to 1.0)	0.0 (0.0 to 1.0)	0.0 (0.0 to 0.0)	0.0 (−1.0 to 0.0)	0.59	0.63	0.25	0.26
Evoked pain	0.0 (−0.3 to 0.0)	0.0 (0.0 to 0.7)	0.0 (−0.1 to 0.5)	0.0 (0.0 to 0.0)	0.19	0.95	0.66	0.50
Paresthesia/dysesthesia	0.0 (0.0 to 1.0)	0.5 (0.0 to 1.0)	0.8 (−0.1 to 1.8)	0.0 (−0.5 to 0.0)	0.53	0.15	0.65	0.02
painDETECT	0.0 (0.0 to 2.0)	0.0 (0.0 to 2.0)	0.5 (−2.2 to 3.0)	0.0 (−1.0 to 1.0)	0.75	0.68	0.78	0.37
BDI	3.0 (0.0 to 4.0)	0.0 (−2.0 to 0.0)	1.0 (−0.2 to 3.5)	0.0 (−2.0 to 0.0)	0.12	0.22	0.93	0.64
PSQI	0.0 (0.0 to 0.0)	0.0 (−1.0 to 0.0)	0.5 (−0.2 to 2.2)	0.0 (0.0 to 2.0)	0.96	0.83	0.43	0.27
WHOQOL-BREF	0.0 (0.0 to 2.0)	0.0 (−5.0 to 0.0)	0.0 (−1.5 to 3.2)	0.0 (−1.0 to 4.0)	0.06	0.91	0.99	0.31

Change scores were calculated from the start of each treatment period (T0) to the 1-week (T1) and 4-week (T2) assessments and are expressed so that positive values indicate improvement (T0 minus follow-up for NPSI, painDETECT, BDI and PSQI; follow-up minus T0 for WHOQOL-BREF). [a] Mann–Whitney U test; modality-matched comparison between independent participants in the active and sham groups. [b] Wilcoxon signed-rank test; within-participant comparison between modalities in the same randomized group (active rTMS vs. active tDCS, n = 12 pairs; sham rTMS vs. sham tDCS, n = 13 pairs). All analyses were exploratory, and p values are not adjusted for multiple comparisons. IQR, interquartile range; NPSI, Neuropathic Pain Symptom Inventory; BDI, Beck Depression

Inventory; PSQI, Pittsburgh Sleep Quality Index; WHOQOL-BREF, World Health Organization Quality of Life-BREF; rTMS, repetitive transcranial magnetic stimulation; tDCS, transcranial direct current stimulation.
